# Changing the Face of Diabetic Care with Haptoglobin Genotype Selection and Vitamin E

**DOI:** 10.5041/RMMJ.10047

**Published:** 2011-04-30

**Authors:** Nina S. Levy, Andrew P. Levy

**Affiliations:** Department of Anatomy, Bruce Rappaport Technion Faculty of Medicine, Technion Israel Institute of Technology, Haifa, Israel

**Keywords:** Haptoglobin, diabetes, genotype, cardiovascular disease, vitamin E

## Abstract

Research over the past 10 years in our laboratory has led to two major findings. The first is that haptoglobin (Hp) genotype can predict the risk of developing vascular complications in individuals with diabetes mellitus (DM), and the second, more far-reaching discovery, is that vitamin E treatment can significantly reduce vascular complications in individuals with DM and the Hp 2-2 genotype. The former finding has been well documented in numerous studies which included over 50,000 patients of diverse geographical and ethnic backgrounds. The latter discovery is more recent and less well accepted by the medical community due to confounding reports over the past 30 years regarding the efficacy of vitamin E treatment for vascular disease. We propose that the benefit of vitamin E treatment was not obvious in earlier studies due to the absence of any genetic basis for patient selection. Our studies dividing DM individuals into vitamin E treatment subgroups based on Hp genotype show a clear benefit for individuals of the Hp 2-2 genotype, while patients carrying the other two Hp genotypes are not affected or may be adversely affected by receiving vitamin E. These findings may explain the overall lack of benefit seen in previous vitamin E studies and emphasize the importance of carefully selecting which patients should receive vitamin E therapy. The pharmacogenomic paradigm discussed in this review potentially could result in a dramatic improvement in the health of millions of individuals worldwide using a treatment that is both accessible and affordable to all.

## INTRODUCTION

Haptoglobin (Hp) was first described as a hemoglobin (Hb)-binding protein present in serum by Polonovski and Jayle in 1938 when they observed that the addition of Hb to serum resulted in an increase in the peroxidase activity of the Hb.^1^ These authors characterized the protein as an alpha-2 glycoprotein and gave the protein the name haptoglobin.^2^ In 1955 Smithies provided a molecular explanation for the apparent heterogeneity in Hp protein molecules purified from different individuals.^3^ Smithies described the separation by gel electrophoresis of Hp proteins into three distinct patterns which he attributed to a genetic polymorphism.^4^

**Figure 1:**
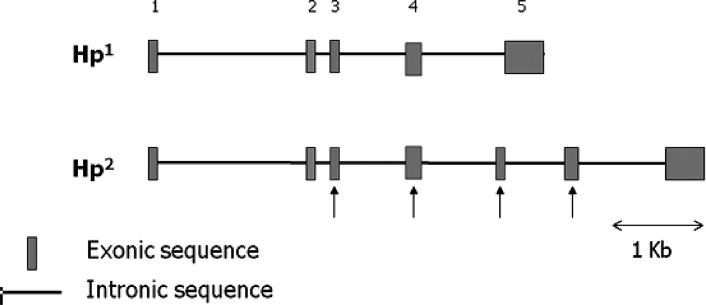
Genomic structure of Hp alleles 1 and 2. Filled in boxes represent exonic sequences, dark lines represent intronic sequences. Hp 1 contains 5 exons. The Hp2 allele evolved from the Hp 1 allele following duplication of exons 3 and 4. Arrows pointing upward identify duplicated exons. Figure included with permission from Mary Ann Liebert, Inc. publishers (Levy et al, Antioxid Redox Signal 2010; 12:293–304).

Subsequent cloning of the Hp gene locus revealed the presence of two alleles in man, denoted Hp 1 and Hp 2.^5^ In rodents and most other mammals there exists one Hp allele containing five exons, known as the Hp 1 allele (Figure 1). The transcript is translated into a single polypeptide chain which is then cleaved into a 45 kD beta chain and a 9 kD alpha chain (called alpha-1). The alpha and beta chains are disulfide linked and form the Hp monomer.

In humans the Hp 1 allele is considered to have undergone a duplication event of exons 3 and 4, creating a seven-exon Hp 2 allele which exists in a balanced polymorphism with the Hp 1 allele. The Hp 2 protein has the same size beta chain and a larger 16 kD alpha chain (called alpha-2) due to the duplication event. The Hp 1/2 polymorphism results in three genotypes present in the human population: Hp 1-1, Hp 2-1, and Hp 2-2. Allele frequencies vary worldwide; however, due to early positive selective pressure for the Hp 2 allele, the Hp 2-2 and Hp 2-1 genotypes account for approximately 36% and 48% of the population, respectively, while the Hp 1-1 genotype is found less frequently in approximately 16% of the population.^6^

**Figure 2:**
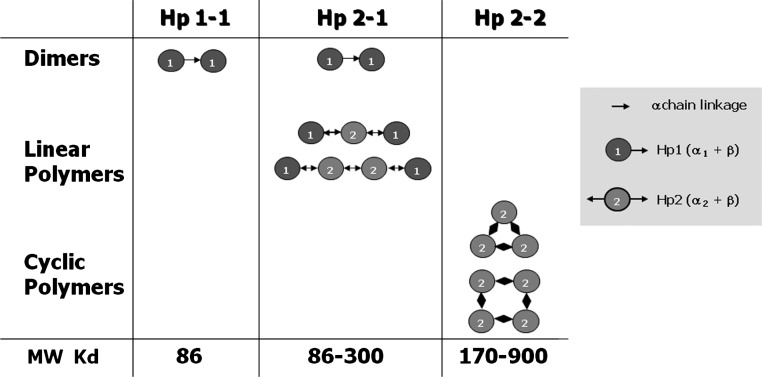
Polymeric structure of protein products resulting from the three Hp genotypes: Hp 1-1, Hp 2-1, and Hp 2-2. All of the subunits in the polymers are disulfide bond linked through the alpha chain. Individuals homozygous for the Hp 1 allele form exclusively dimers containing two Hp 1 subunits. Heterozygotes form linear polymers containing a variable number of internal Hp 2 subunits flanked by two Hp 1 subunits at either end. Individuals homozygous for the Hp 2 allele form cyclic polymers of varying numbers of Hp 2 subunits. The molecular weight of the polymers is smallest for Hp 1-1 at 80 kD, larger for Hp 2-1 ranging from 86–300 kD, and largest for Hp 2-2 ranging from 170–900 kD. Figure included with permission from Mary Ann Liebert, Inc. publishers (Levy et al, Antioxid Redox Signal 2010; 12:293–304).

The most significant consequence of the duplication event which led to the formation of the Hp 2 allele is the addition of a cysteine residue in the alpha-2 chain. This cysteine participates in the formation of disulfide linkage between two Hp monomers. Each Hp 2 monomer has the ability to form two intermolecular disulfide bonds. This single feature leads to a profound difference in polymeric structure. While Hp 1-1 individuals form Hp 1 dimers exclusively, Hp 2-1 individuals form linear polymers made up of varying numbers of Hp 2 subunits (average two) flanked by two Hp 1 monomers at either end. Hp 2-2 individuals have yet a different arrangement in that the Hp 2 monomers form cyclic polymers containing an average of four Hp 2 subunits (Figure 2).

## FUNCTIONAL ASPECTS OF HAPTOGLOBIN

In 1957 it was first demonstrated that the Hp–Hb complex cannot pass through the glomerular filter, thereby implicating Hp as a major mediator of Hb (as well as iron) conservation.^7^ When Hp is depleted as a result of hemolysis^8^ or in Hp knockout mice,^9^ Hb is found to accumulate in the kidney and is secreted in the urine. Therefore, an additional role of Hp is to prevent renal damage.^10^ The protective function of Hp is thought to lie in its ability to stabilize heme iron within Hb and thereby prevent subsequent iron-induced oxidative damage. This hypothesis is supported by studies showing that Hp inhibits transfer of heme from Hb to low-density lipoprotein (LDL), thereby preventing LDL oxidation.^11^

Hp acts as a bacteriostatic agent by restricting access of bacteria to Hb-derived iron which is critical for bacterial growth. As infectious disease was likely the most important selective pressure early in human evolution, the fact that the Hp 2 allele spread so rapidly worldwide is highly supportive of the notion that the Hp 2 allele provides selective resistance to foreign pathogens. One example of this phenomenon has been demonstrated for the Streptococcus bacterium. The T antigen (specifically T4) on the coat of Streptococcus binds Hp. Polymeric Hp 2-2 molecules agglutinate and cause clumping of the Streptococcus organisms, thereby markedly inhibiting their growth.^6^

Hp non-complexed to hemoglobin has been found to have angiogenic activity. For example, patients with systemic vasculitis were shown to contain an angiogenic factor in their serum which was identified as Hp. Purified Hp was subsequently shown to be angiogenic in two *in-vivo* models.^12^ Results from animal models of myocardial ischemia have implicated the Hp protein in coronary collateral development.^13^ The Hp 2-2 protein appears to have a greater potency than Hp 1-1 as deduced from genotype studies of patients with peripheral arterial occlusive disease.^14^ It was this angiogenic function of Hp which ultimately led to our studies linking Hp to diabetes, as will be described below.

## LINKING HAPTOGLOBIN TYPE TO DIABETES MELLITUS COMPLICATIONS

The discovery of a connection between Hp type and diabetic complications was in retrospect entirely serendipitous, based on a hypothesis that was subsequently proven to be incorrect. Research in our laboratory at the time of the initial discovery about 13 years ago was focused on the hypoxic induction of vascular endothelial growth factor (VEGF) and its role in coronary collateral vessel formation. Studies showed that those individuals with more extensive collateral formation had higher induction of VEGF in response to hypoxia in monocyte cultures.^15^ However, it was not clear why monocytes from certain individuals were able to induce VEGF to a high degree while monocytes from other individuals were not. We hypothesized that a genetic polymorphism could be responsible for the interindividual differences seen in the hypoxic induction of VEGF.

A study published at that time reported that individuals of the Hp 2-2 genotype had an increased incidence of peripheral arterial occlusive disease compared to Hp 1-1 individuals,^13^ yet they were able to walk further, suggesting that Hp 2-2 might be stimulating more peripheral collaterals via up-regulation of VEGF. We measured the hypoxic inducibility of VEGF as well as Hp genotype in monocytes of individuals suffering from peripheral vascular disease but were unable to find any significant relationship.

Using a second approach, we reasoned that perhaps this relationship might be evident *in vivo* by examining disease states in which VEGF was implicated, such as restenosis after angioplasty and diabetic vascular disease. Our analysis of a relatively small cohort revealed the existence of a highly significant relationship between the development of vascular complications of diabetes and the Hp genotype. It was found that for three independent end-points, restenosis after angioplasty, retinopathy, and nephropathy, none of the diabetic patients with the Hp 1-1 genotype developed disease, while 30%–40% of the patients with the other two genotypes developed disease. Table 1 lists a number of important longitudinal studies which demonstrated that diabetic individuals of the Hp 2-2 genotype have on average about a 3-fold (1.5–5) increased risk of developing vascular complications than do Hp 1-1 diabetic individuals.^16^–^22^ Hp 2-1 diabetics were shown to have an intermediate risk. The increase in risk for diabetic Hp 2-2 individuals was shown to be true for type I diabetics as well as type II diabetics.^22^ In all of the analyses listed there were no gender differences seen in Hp 2-2-associated risk. Two studies (Strong Heart Study^16^ and the Rambam post-MI study^18^) showed that Hp genotype is not predictive of vascular complications in non-diabetic individuals.

## HAPTOGLOBIN 2-2 PROTEIN IS AN INFERIOR ANTIOXIDANT

Serum haptoglobin binds free Hb released into plasma due to red blood cell senescence or hemolysis. The binding constant for Hp–Hb is on the order of 10^15^, and that fact, along with the high concentration of Hp in serum (0.3 mg/mL up to 3 mg/mL), effectively eliminates the presence of any free Hb in the blood-stream. The binding of Hb to Hp is critical for sequestering the pro-oxidant iron present in Hb and preventing its participation in oxidative reactions such as the hydroxyl radical-forming Fenton reaction. We sought to determine whether the different Hp types might have dissimilar antioxidant activity which could account for why Hp genotype was predictive of diabetic complications. This seemed like a plausible hypothesis since it was well accepted that oxidative stress plays a major role in the development of diabetic complications.

Several studies support the notion that the Hp 2-2 polymer is a less powerful antioxidant than the Hp 1-1 dimer. First, it was shown that Hp 2-2 individuals have a lower level of serum vitamin C compared to Hp 1-1 individuals.^23^ This result suggested that the iron present in the Hp 2-2–Hb complexes is more reactive, resulting in a decrease in vitamin C levels. Second, purified Hp 2-2–Hb complexes were found to oxidize linoleic acid and LDL to a greater degree than Hp 1-1–Hb complexes.^11^,^24^ Third, Hp 2-2 diabetic individuals were found to have a higher level of labile redox-active non-transferrin bound plasma iron than Hp 1-1 diabetics.^25^

## ATHEROSCLEROTIC PLAQUES AND KIDNEYS OF HP 2-2 DM MICE AND HUMANS CONTAIN INCREASED AMOUNTS OF IRON

A number of human study findings have been recapitulated in our transgenic diabetic mouse model. Wild-type mice contain only the Hp 1 allele. We created a mouse Hp 2 allele by producing an intragenic duplication of exons 3 and 4 of the mouse Hp 1 allele and inserted this mouse Hp 2 allele at the mouse Hp genetic locus by targeted genetic recombination. We have demonstrated that Hp 2-2 diabetic mice develop more severe nephropathy,^26^ retinopathy,^27^ and heart damage following experimental coronary artery occlusion^28^ than their Hp 1-1 counterparts. Diabetic Hp 2-2 transgenic mice crossed into an Apo E-deficient background were shown to develop plaques in the brachiocephalic artery with a greater degree of inflammation and oxidative stress. Perl’s iron staining of mouse atherosclerotic plaque and kidney tissue showed increased iron in diabetic Hp 2-2 mice.^29^–^30^ This increase in plaque iron was also demonstrated in human Hp 2-2 DM plaques.^31^ Electron micrographs of plaques and renal tissue from Hp 2-2 diabetic mice showed an increase in iron deposits specifically in the lysosome (unpublished results). These results suggest that Hp 2-2–Hb-derived iron accumulation inside the lysosome may be responsible for the pathophysiology associated with the Hp 2-2 genotype.

## CLEARANCE OF HP 2-2–HB COMPLEXES FROM SERUM AND TISSUES IS IMPAIRED

Hp–Hb complexes have been shown to bind with high specificity and affinity to the CD163 receptor present on monocytes and macrophages in humans. Hp–Hb complexes are believed to be cleared from the blood-stream and from tissues by this receptor. Among DM individuals, the Hp 2-2 genotype was associated with a greater than 50% decrease in the percentage of peripheral blood monocytes expressing CD163 and an increase in plasma-soluble CD163, suggesting an increase in membrane shedding of the CD163 receptor in Hp 2-2 diabetics.^32^*In-vitro* studies using radiolabeled complexes added to cells stably transfected with the human CD163 receptor indicated that Hp 1-1–Hb complexes are taken up at a faster rate than Hp 2-2–Hb complexes.^33^ Taken together, these results support the notion that a CD163-dependent mechanism leads to less efficient clearance of Hp 2-2–Hb complexes from the blood-stream and tissues as compared with Hp 1-l–Hb complexes.

We have attempted to recapitulate the role of CD163 in the altered clearance of Hp 2-2–Hb complexes in our mouse transgenic model. The mouse CD163 receptor is induced by glucocorticoids and is expressed on peripheral blood monocytes and macrophages, similar to the human receptor.^34^ However, we have been unable to show specific binding of human or mouse Hp–Hb complexes to cells stably transfected with the mouse CD163 receptor. In addition, mouse macrophages do not respond by secreting interleukin 10 (IL-10) in response the Hp–Hb complexes as do human macrophages. To our knowledge, there have been no reports in the literature of mouse CD163 receptor binding to Hp–Hb complexes.

Interestingly, *in-vivo* clearance studies showed that following injection of radiolabeled Hp–Hb complexes into diabetic mice transgenic for Hp 2, Hp 2-2–Hb complexes demonstrated a 5.7-fold increase in clearance time compared to Hp 1-1–Hb complexes.^35^ Our anomalous results regarding the mouse CD163 receptor suggest either that mice have a different receptor for Hp–Hb or that the mechanism responsible for the differences seen in clearance of Hp–Hb complexes is not related to CD163 receptor binding and lies elsewhere in the clearance pathway.

## HDL FUNCTION IS IMPAIRED IN HP 2-2 DM INDIVIDUALS

It has been reported that Hp binds to high-density lipoprotein (HDL) via the Apo Ai protein. Our laboratory has shown that more Hb was associated with HDL from diabetic individuals of the Hp 2-2 genotype, possibly due to the decreased clearance of Hp 2-2–Hb complexes from the blood-stream and the accumulation of Hp 2-2–Hb levels.^36^ The presence of redox-active ion bound to the HDL particle suggested that neighboring proteins and lipid components may undergo oxidation and functional alteration. Lecithin cholesterol acyl transferase (LCAT), an HDL-associated protein, is critical for the esterification of HDL cholesterol and the maturation of HDL. We have found that LCAT activity is markedly reduced in the serum of Hp 2-2 DM individuals as compared to Hp 1-1 DM individuals, with an intermediate level of activity in Hp 2-1 DM individuals.^37^ In addition, experiments which utilize the ability of HDL particles to promote reverse cholesterol transport *in vitro* showed that serum from diabetic Hp 2-2 individuals was less active than serum from diabetic Hp 1-1 individuals. These results showing altered HDL structure and function in Hp 2-2 diabetics have been recapitulated using serum from diabetic transgenic mice.^37^

## PREVENTION OF DM COMPLICATIONS IN HP 2-2 DM INDIVIDUALS WITH VITAMIN E

The studies presented above have suggested that diabetic Hp 2-2 individuals suffer from increased oxidative stress over and above that experienced by diabetic Hp 1-1 individuals due to the impaired antioxidant function of the Hp 2-2 polymer and the increased presence of reactive iron in these individuals. It was reasonable therefore to propose that providing Hp 2-2 DM individuals with antioxidants might be helpful in reducing the burden of disease in this population. Vitamin E is a well known antioxidant which stops the production of reactive oxygen species formed when fat undergoes oxidation. Although the protective effects of vitamin E in previous clinical studies have been controversial, we reasoned that a beneficial effect may become apparent in a subgroup of patients suffering from excessive oxidative stress, such as the Hp 2-2 diabetic group. In addition, vitamin E was considered a strong candidate for antioxidant therapy since it is inexpensive, has been widely used in the past with few adverse effects, and is known to be present in high concentrations in the lysosomal membrane, the site of iron accumulation (the critical oxidant) in our studies.

Three complementary approaches have been taken to explore this possible pharmacogenomic relationship between the Hp type and vitamin E. First, serum samples from previous clinical studies in which patients were treated with vitamin E in order to prevent vascular disease were analyzed for Hp genotype. Second, a prospective study was undertaken in Hp 2-2 diabetic patients to determine efficacy of vitamin E treatment in preventing cardiovascular complications. Third, vitamin E was given to mice in our Hp transgenic mouse model of diabetes to further corroborate efficacy of treatment.

Archived serum samples from the Heart Outcomes Prevention Evaluation (HOPE) study were analyzed for Hp genotype and correlated with the occurrence of non-fatal myocardial infarction (MI), stroke, and cardiovascular disease (CVD) death in patients with type 2 diabetes. The results showed that Hp 2-2 diabetics who received vitamin E had a 31% reduction in the composite primary end-point of MI, stroke, and CVD death compared to placebo controls (and an approximately 20% decrease in overall mortality). In the Hp 2-1 vitamin E-treated group there was an increase of 11% in the composite outcome.^20^ A similar retrospective analysis of the Women’s Health Study (WHS) showed that Hp 2-2 diabetics given vitamin E suffered 15% less CVD and 8% less mortality. In WHS Hp 2-1 and Hp 1-1 diabetics receiving vitamin E showed an increase in CVD of 25% and 19%, respectively, as well as an increase in mortality of 39% and 9%, respectively.^21^

**Figure 3:**
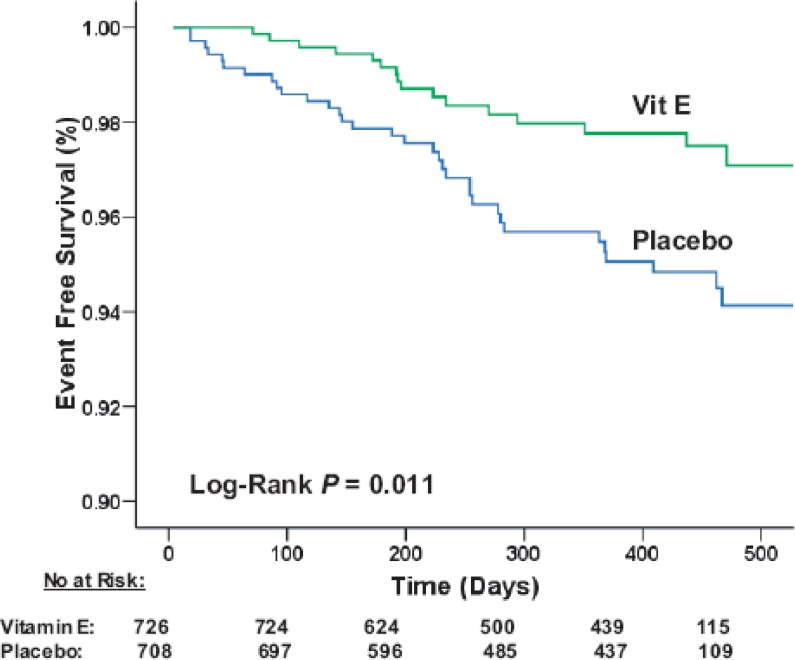
Kaplan-Meier plot of the composite end point in Hp 2-2 DM individuals allocated to vitamin E or placebo. Events are CV death, MI, or stroke. There were 726 Hp 2-2 individuals allocated to vitamin E and 708 Hp 2-2 individuals allocated to placebo. As a reflection of the 18-month window during which participants entered the study (time 0 being the day of Hp typing) and the early termination of the study not all participants were in the study for the same duration. This is reflected in the abscissa where the number of individuals in the study (the number at risk) for a given duration is provided. There were a total of 16 patients (2.2%) who had events in the vitamin E group and 33 patients who had events in the placebo group (4.7%). There was a significant decrease in the composite end point in the vitamin E group compared with the placebo group (Hazard Ratio 0–47 [95% Confidence Interval 0.27 to 0.82], *P*=0.01 by log-rank). Figure included with permission from American Heart Association. (Milman et al.^19^).

Israel Cardiovascular Events Reduction with Vitamin E (ICARE) was a prospective trial in which diabetic patients were tested for the Hp 2-2 genotype and then randomly assigned to either treatment with vitamin E or placebo. Dosage was based on a retrospective analysis of vitamin E levels which were cardioprotective in the Hp 2-2 subgroup in the HOPE study. In the vitamin E-treated group there was a 54% decrease in MI, stroke, and CVD death (Figure 3). After vitamin E treatment was discontinued the rate of MI was increased 4.5-fold (0.4% on vitamin E, 1.8% after vitamin E) compared to the treatment period.^19^ Overall the results from the retrospective HOPE and WHS analysis as well as the prospective ICARE studies are consistent with the notion that vitamin E is beneficial for Hp 2-2 diabetics. However, the same cannot be said for diabetics of the non-Hp 2-2 genotype. In fact, vitamin E treatment appears to be harmful in some cases, particularly in the Hp 2-1 subgroup.

**Figure 4:**
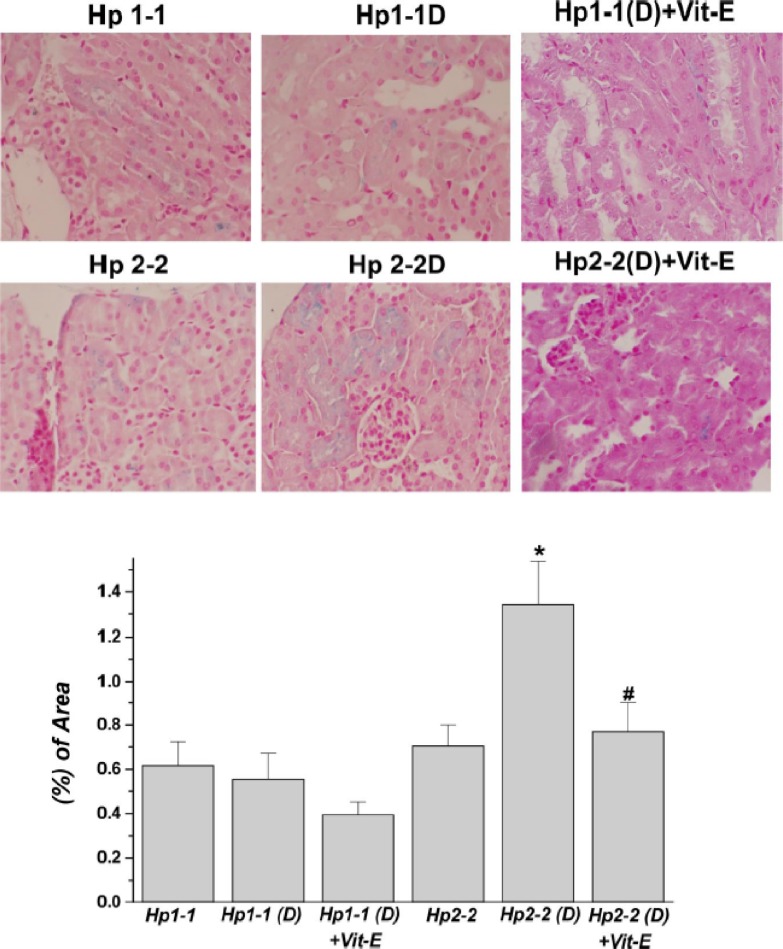
Increased renal iron deposition in the proximal tubule of Hp 2-2 mice with Diabetes Melitus (DM). Perl’s iron stain was used to localize iron in paraffin-embedded kidney sections in Hp 1-1 and Hp 2-2 mice with and without DM. Arrow indicates iron-induced stain in blue (×400 magnification) located within proximal tubular cells. There was a significant increase in iron staining in the renal tissue of Hp 2-2 DM (D) vs. Hp 1-1 DM (D) and Hp 2-2 non-DM mice (*P* < 0.001; *n* = 6 animals for each group). *Hp 2-2 (D) vs. Hp2-2. # Hp 2-2 (D) + Vit-E vs. Hp 2-2 (D). Figure included with permission from American Physiological Society. (Nakhoul et al.^26^).

Studies using diabetic transgenic mice showed that vitamin E treatment decreased the severity of kidney disease as well as the amount of iron in the proximal tubules (Figure 4) in Hp 2-2 mice compared to non-treated Hp 2-2 mice.^26^ In addition, HDL function (assessing the ability to promote cholesterol efflux from macrophages loaded with cholesterol *in vitro)* using serum from Hp 2-2 mice (or humans) treated with vitamin E was improved over that from non-treated mice.^28^ Purified HDL from vitamin E-treated Hp 2-2 mice was also shown to contain fewer lipid peroxides than non-purified HDL from non-treated mice. These studies support the beneficial role of vitamin E in the setting of diabetes in Hp 2-2 individuals and should allow for a more detailed analysis of the effects of vitamin treatment on disease progression in diabetic individuals of Hp 2-1 and Hp 1-1 genotypes.

## FUTURE DIRECTIONS: TRANSLATING RESEARCH FINDINGS INTO THE CLINIC

In order to translate these findings relating the Hp type, vitamin E, and diabetic vascular disease into the medical clinic, we recognize the need to surmount three major hurdles. The first is the development of a rapid and affordable assay for the determination of Hp genotype in diabetic individuals. To date, gel electrophoresis and PCR have been the most commonly used methods for genotype analysis. While both are highly accurate, they are relatively time-consuming processes, requiring 3–4 hours. Our laboratory has been involved in developing an ELISA-based assay for detecting Hp genotype. This assay takes advantage of differences in the polymeric nature of the different Hp types and uses the same Hp 2 specific monoclonal antibody for detection and capture. In this assay, one obtains a very low signal for Hp 1-1, an intermediate signal for Hp 2-1, and a high signal for Hp 2-2. The ELISA has been validated in over 5,000 individuals and compared with serum gel electrophoresis with greater than 98% correspondence in Hp assignment. Commercialization of this ELISA assay should allow for the easy, rapid, inexpensive, and routine determination of Hp genotype in diabetic individuals.

A second line of research is directed towards conducting additional, larger-scale prospective studies of approximately 3,000 Hp 2-2 diabetics randomized to treatment with vitamin E or placebo. These studies will be carried out separately in the US and in Europe and should provide the necessary statistical significance required for establishing conclusive treatment guidelines. Funding for these types of studies is difficult to attract due to the lack of interest on the part of drug companies owing to the non-lucrative nature of vitamin E, and therefore non-profit governmental-based funding agencies represent the only viable possible source of support.

The third line of research involves deciphering the mechanism by which Hp 2-2 exacerbates vascular complications in the setting of diabetes and, no less important, how vitamin E helps Hp 2-2 individuals while it may harm non-Hp 2-2 individuals. The former question will be addressed by taking a closer look at how iron is sequestered inside the cell. Specifically we plan to use radiolabeled iron to follow iron accumulation and to study the effects of Hp–Hb-derived iron on lysosome function. The latter question is more difficult to address, as it would be unethical to design prospective studies treating Hp 2-1 individuals with potentially harmful vitamin E. Therefore, transgenic studies will be used to address this question, as well as *in-vitro* studies using purified Hp 2-1 complexes. We hypothesize that under certain conditions, apparently afforded by the Hp 2-1 polymer, vitamin E is acting as a pro-oxidant instead of as an antioxidant.

## SUMMARY

It is our belief that vitamin E will prove to be highly effective in treating a significant subgroup of diabetic patients. This new advance has the potential to reduce health costs considerably and also improve quality of life for many diabetics. However, the precise mechanism by which Hp genotype determines diabetic vascular complications remains to be elucidated. Further research in this direction will hopefully lead to a more precise and comprehensive algorithm for treating diabetics of all the haptoglobin genotypes.

## Figures and Tables

**Table 1:** Patient studies which demonstrated that Hp 2-2 diabetics are at a higher risk for the development of vascular disease than Hp 1-1 diabetics.

**STUDY**	**OUTCOME**
Strong Heart Study^16^	3–5 fold increase in CVD* in Hp 2-2
Munich Percutaneous Coronary Intervention (PCI) Study^17^	2.3 fold increase in MI* in Hp 2-2
Rambam post MI study^18^	3–5 fold increase in death or congestive heart failure in Hp 2-2
Israel Cardiovascular Reduction Events (ICARE)^19^	2.3 fold increase in CVD in Hp 2-2
Heart Outcomes Prevention Evaluation (HOPE)^20^	2.3 fold increase in CVD in Hp 2-2
Womens’ Health Study (WHS)^21^	1.5 fold increase in CVD in Hp 2-2
Epidemiology of Diabetes Complications (EDC)^22^	2.1 fold increase in CVD in Hp 2-2

CVD, cardiovascular disease; Hp, Haptoglobin; MI, myocardial infarction.

## Abbreviations:

CD163: cluster of differentiation 163
CVD: cardiovascular disease
DM: diabetes mellitus
ELISA: enzyme-linked immunosorbent assay
Hb: hemoglobin
HDL: high-density lipoprotein
HOPE: Heart Outcomes Prevention Evaluation
Hp: haptoglobin
ICARE: Israel Cardiovascular Events Reduction with Vitamin E
IL-10: interleukin 10
LCAT: lecithin cholesterol acyl transferase
LDL: low-density lipoprotein
MI: myocardial infarction
VEGF: vascular endothelial growth factor
WHS: Women’s Health Study.
